# Managing Catheter Knotting in Pulse‐Select Pulsed Field Ablation: Mechanisms and a Practical Strategy for Resolution

**DOI:** 10.1002/joa3.70250

**Published:** 2025-12-11

**Authors:** Masateru Takigawa, Ryosuke Kato, Masaki Honda, Iwanari Kawamura, Ryo Tateishi, Miho Negishi, Kentaro Goto, Takuro Nishimura, Kazuya Yamao, Susumu Tao, Shinsuke Miyazaki, Tetsuo Sasano

**Affiliations:** ^1^ Department of Cardiovascular Medicine Institute of Science Tokyo Tokyo Japan; ^2^ Advanced Arrhythmia Research Institute of Science Tokyo Tokyo Japan

**Keywords:** atrial fibrillation, complication, knotting, pulsed field ablation, PulseSelect

## Abstract

**Background:**

Pulsed field ablation (PFA) using the PulseSelect catheter is effective for atrial fibrillation but may occasionally result in catheter deformation or knotting.

**Methods:**

We analyzed 25 PulseSelect catheters used for AF ablation to identify knotting mechanisms. Two reproducible knot types were observed. A resolution strategy was tested: retraction to the sheath tip, wire withdrawal, and gradual advancement with or without rotation.

**Results:**

The knot was resolved in 24 catheters (96%), with 21 (84%) resolved on the first attempt. One severe type 1 knot was resistant but safely removed.

**Conclusions:**

A structured, non‐forceful approach utilizing the catheter's elasticity effectively resolves knotting without rendering the catheter unusable.

## Graphical abstract

This study describes two types of PulseSelect catheter knotting and demonstrates a simple, non‐forceful resolution strategy. This technique, which utilizes the catheter's inherent elasticity, successfully resolved Type 1 knotting in 96% and Type 2 knotting in 100%, enhancing procedural safety without damaging the device.

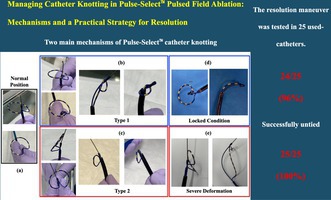

## 1 Introduction

Pulsed field ablation (PFA) is an innovative technology offering selective myocardial ablation [1] with minimal damage to collateral tissues compared to conventional radiofrequency energy. Its safety and efficacy have been demonstrated in preclinical and clinical studies [2, 3, 4, 5]. However, catheter designs differ among manufacturers, potentially influencing efficiency and safety. The PulseSelect system (Medtronic Inc., Minneapolis, MN, US), approved by the FDA in December 2023, has shown favorable outcomes in PFA [1]. However, its unique design may lead to complications such as knotting, twisting, and deformation, as reported in the MAUDE database [6]. This report documents 25 cases of material deformation, including catheter tip knotting, array inversion/twisting, and kinking, with six involving insulation breaches or exposed wires. Five cases required catheter replacement, and one needed additional ablation with a new catheter. There were seven entrapment cases: six with the guidewire and one with the slider tool. Of these, four were due to deformation, one to contamination, and two had unknown causes. While none of the events led to procedure termination or direct patient harm, such issues may increase the risk of further complications. This study investigates the mechanisms of catheter knotting and proposes a systematic strategy for resolution.

## 2 Methods

### 2.1 Two Types of Knot Formation

Knot formation can occur during catheter manipulation when the wire is withdrawn and the distal tip presses against tissue. This may cause the nose portion to shift away from the center of its circular shape. If the wire is reinserted while the tip remains misaligned and the catheter is retracted into the sheath, a knot may form.

The knot type depends on the direction of nose deviation. Two distinct types of deformation have been observed. The first involves inward displacement of the PulseSelect catheter nose into the loop, resulting in intra‐loop knotting (b). The second involves outward deviation of the nose along the guidewire, leading to extra‐loop knot formation (c).

### 2.2 Strategy for Knot Removal

If resistance is encountered while retracting the catheter into the sheath, forceful pulling should be avoided to prevent further complications or deformation. Upon detecting a knot at the catheter tip, the following steps are advised: first, retract the catheter to the sheath tip; then withdraw the internal wire; finally, slowly advance the catheter out of the sheath (see Videos 1 and 2).

**VIDEO 1:** The strategy used to remove a type 1 knot, in which the catheter tip is entrapped within the inner loop. Video content can be viewed at https://onlinelibrary.wiley.com/doi/10.1002/joa3.70250.

**VIDEO 2:** The technique applied to resolve a type 2 knot, where the catheter tip is displaced externally beyond the loop via the guidewire. Video content can be viewed at https://onlinelibrary.wiley.com/doi/10.1002/joa3.70250.

**FIGURE 1:**
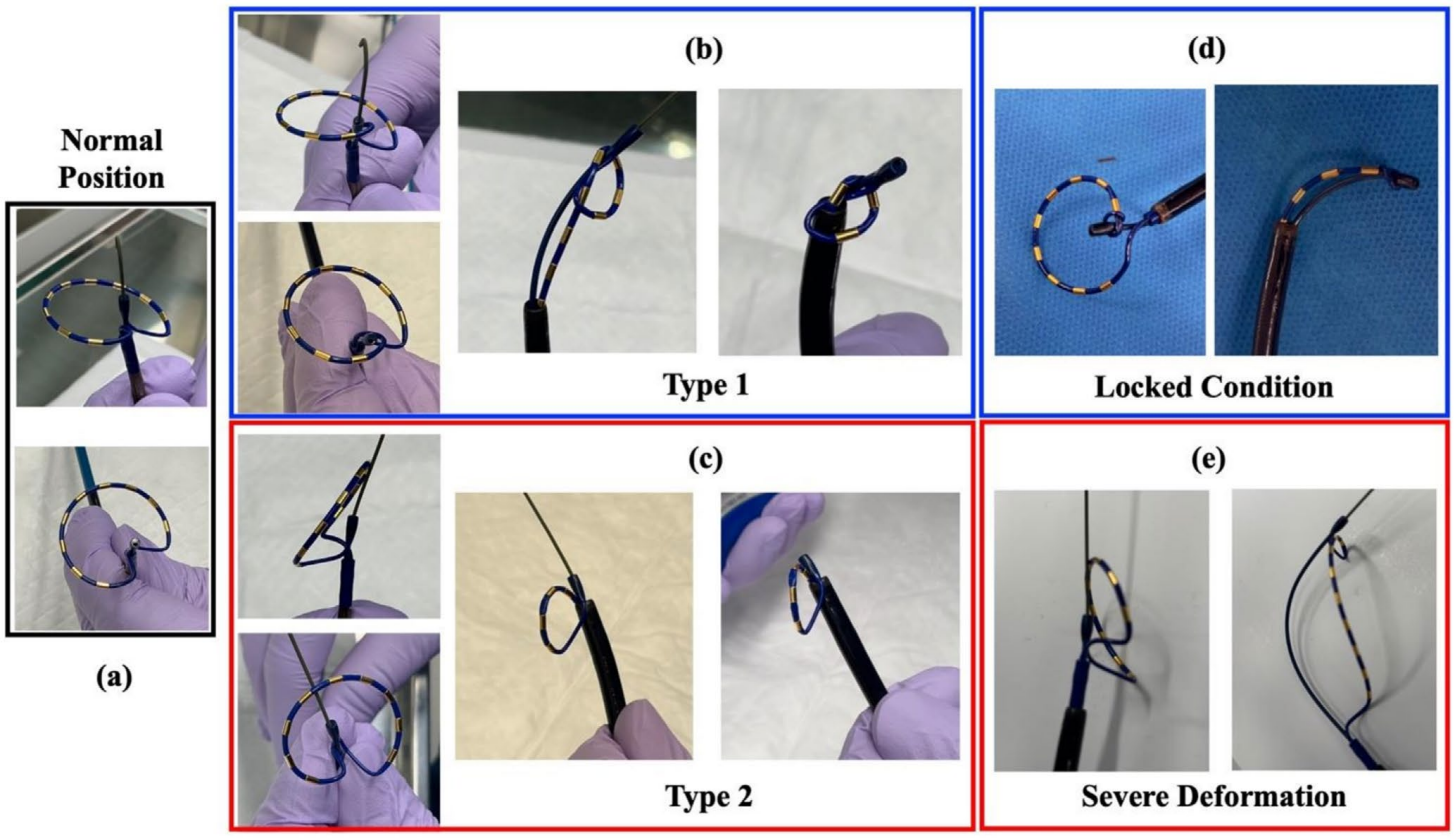
Normal position of the catheter (a). Two distinct types of deformation have been observed. The first involves inward displacement of the PulseSelect catheter nose into the loop, resulting in intraloop knotting (b). The second involves outward deviation of the nose along the guidewire, leading to extraloop knot formation (c). Forcibly pulling the catheter from the sheath may further tighten the knot, resulting in a locked configuration (d) or unrecoverable severe deformation (e).

In most cases, this maneuver lets the displaced nose return to its central alignment, resolving the knot. If not successful initially, repeated attempts may still correct it. However, when the nose is severely displaced due to excessive manipulation, resolution becomes difficult (Figure 1d,e). Forcibly pulling the catheter from the sheath may further tighten the knot, resulting in a locked configuration (Figure 1d) or unrecoverable severe deformation (Figure 1e).

### 2.3 Study Methodology

We analyzed 25 PulseSelect catheter and FlexCath Contour sheath sets used for AF ablation as shown in Table 1. Two distinct knot types were intentionally simulated by manipulating the catheter tip. The proposed resolution involved retracting the catheter to the sheath tip, withdrawing the wire, and gradually advancing it from the sheath. The success rate of this approach was evaluated.

**TABLE 1:** Cases treated with PulseSelect catheter and Flexcath Contour sheath sets.

Pt number	Age	Male/Female	PAF/PEF/LSPEF	PVI	LPV	RPV	Posterior LA isolation	Total application number	Number of the attempts until Type 1 knot removal	Forced removal	Number of the attempts until Type 2 knot removal	Forced removal
1	68	M	PAF 2nd	y	15	16	12	43	1	y	1	y
2	68	M	PAF	y	17	19	0	36	3	y	1	y
3	68	M	PEF	y	16	21	16	53	1	y	1	y
4	73	M	PEF 2nd	y	0	0	30	30	1	y	1	y
5	77	M	PEF	y	15	17	12	44	1	y	1	y
6	72	F	PAF	y	20	18	0	38	Not removed	y	1	y
7	55	M	PEF	y	30	19	12	61	1	y	1	y
8	66	F	PEF	y	27	21	0	48	1	y	1	y
9	71	M	PAF 3rd	y	0	0	18	18	1	y	1	y
10	75	F	PAF	y	20	19	0	39	2	y	1	y
11	63	F	PAF	y	24	19	0	43	1	y	1	y
12	75	F	PAF	y	20	20	22	62	1	y	1	y
13	68	M	PEF	y	31	18	19	68	1	y	1	y
14	63	M	PAF	y	18	20	0	38	1	y	1	y
15	68	M	LSPEF	y	21	22	27	70	1	y	1	y
16	75	M	PAF	y	22	21	0	43	1	y	1	y
17	87	M	PAF	y	16	16	0	32	1	y	1	y
18	49	M	PAF	y	20	22	0	42	1	y	1	y
19	66	M	PAF	y	18	25	0	43	1	y	1	y
20	69	M	PAF	y	21	22	0	43	1	y	1	y
21	61	M	PAF	y	19	19	0	38	1	y	1	y
22	49	M	PEF	y	19	25	0	44	1	y	1	y
23	80	M	PAF	y	20	19	0	39	2	y	1	y
24	72	M	PAF	y	19	27	0	46	1	y	1	y
25	75	M	PEF 2nd	y	18	19	0	37	1	y	1	y

Abbreviations: LA, left atrium; LPV, left pulmonary vein; LSPEF, long‐standing persistent atrial fibrillation; PAF, paroxysmal atrial fibrillation; PEF, persistent atrial fibrillation; PVI, pulmonary vein isolation; RPV, right pulmonary vein.

## 3 Results

### 3.1 Clinical Data

This catheter was used in 25 cases for PVI (*N* = 25) and posterior LAI (*N* = 9), in which acute success was achieved. On average, the catheter was used for 43[38–47] applications of energy delivery overall, and 39[37–43] applications specifically for PVI.

### 3.2 Success Rate of This Strategy

Using this method, the knot was successfully removed in 24 (96.0%) out of 25 cases; 21 (84.0%) of them were untied with the first attempt. The one unresolved knot was type 1, with the nose portion strongly bent in the inside direction. Even after applying the method, the nose did not spring back to the center, and repeated attempts failed to resolve the knot.

## 4 Discussion

In this study, we simulated two common PulseSelect catheter deformation patterns and showed that our strategy resolved 96.0% of knots. Despite using the proposed technique, initial resolution failed in 3 of 25 catheters, all employed solely for PVI, suggesting that knot formation may occur independently of procedural complexity. The key principle is to pay attention to the nose deviation during the procedure and avoid applying force when resistance is felt during retraction. Instead, after refracting the catheter to the sheath tip, the internal wire should be withdrawn followed by gradual advancement of the catheter out of the sheath. This leverages the tip's natural tendency to return to its center, effectively untangling the knot in most cases. Forcibly stretching and withdrawing the catheter may further tighten the formed knot, rendering it irreducible [7].

If initial attempts fail, repeated maneuvers with slight rotation may be successful. However, if the nose is excessively displaced and loses its elastic recoil, the knot may become irreducible and this technique ineffective. In such cases, the wire should remain in place, and the catheter should be gently stretched to reduce knot size, allowing careful retrieval into the sheath. However, the catheter may not be reusable due to the extent of deformation.

## 5 Conclusion

This study showed that two common PulseSelect catheter knot types can be resolved safely and effectively using a simple strategy, with a 96.0% success rate. By avoiding excessive force and utilizing the catheter's inherent elasticity, most knots were untied without damage. This method offers a practical solution to improve catheter handling and procedural safety.

## Funding

The authors have nothing to report.

## Conflicts of Interest

Drs. Goto, Takigawa, and Miyazaki belong to the division that receives research endowments from Medtronic Japan, Boston Scientific, Japan Lifeline, and WIN international. Drs. Takigawa, Miyazaki, and Sasano received a lecture fee from Medtronic Japan. The other authors have no conflicts of interest to declare.
